# Supplementary material for: Impacts of propagating, frustrated and surface modes on radiative, electrical and thermal losses in nanoscale-gap thermophotovoltaic power generators
Source: Sci Rep. 2015 Jun 26;5:11626. doi: 10.1038/srep11626 (PMC4481525; doi:10.1038/srep11626)
Supplement: Supplementary Information [file srep11626-s1.pdf]

Supplemental Information: Impacts of propagating, frustrated and surface modes on radiative, electrical and thermal losses in nanoscale-gap thermophotovoltaic power generators

Michael P. Bernardi,[1,a)] Olivier Dupré,[2] Etienne Blandre,[2] Pierre-Olivier Chapuis[2], Rodolphe Vaillon,[2,b)] and Mathieu Francoeur[1,c)]

[1]Radiative Energy Transfer Lab, Department of Mechanical Engineering, University of Utah, Salt Lake City, UT 84112, USA

[2]Université de Lyon, CNRS, INSA-Lyon, UCBL, CETHIL, UMR5008, F-69621 Villeurbanne, France

---

[a)] Electronic mail: michael.bernardi@utah.edu
[b)] Electronic mail: rodolphe.vaillon@insa-lyon.fr
[c)] Electronic mail: mfrancoeur@mech.utah.edu

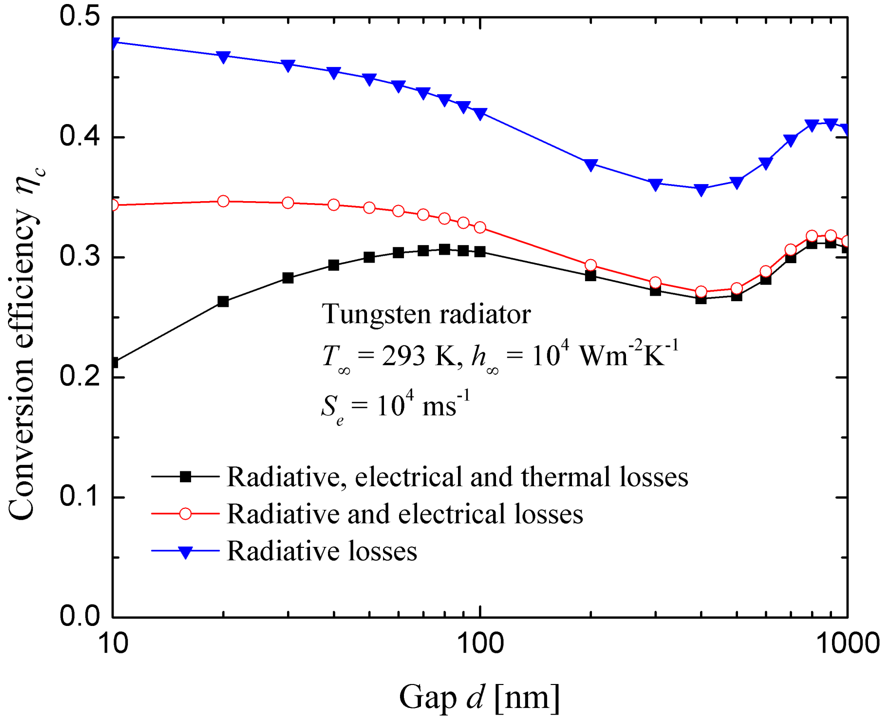

(a)

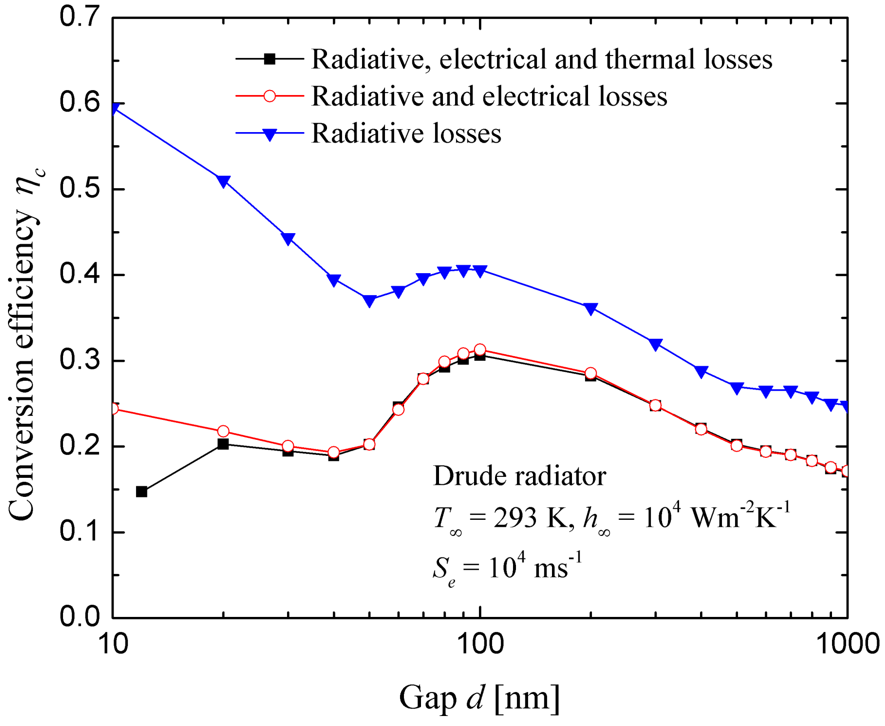

(b)

Figure S.1. Conversion efficiency as a function of the vacuum gap thickness and the type of losses considered: (a) tungsten radiator. (b) Drude radiator.

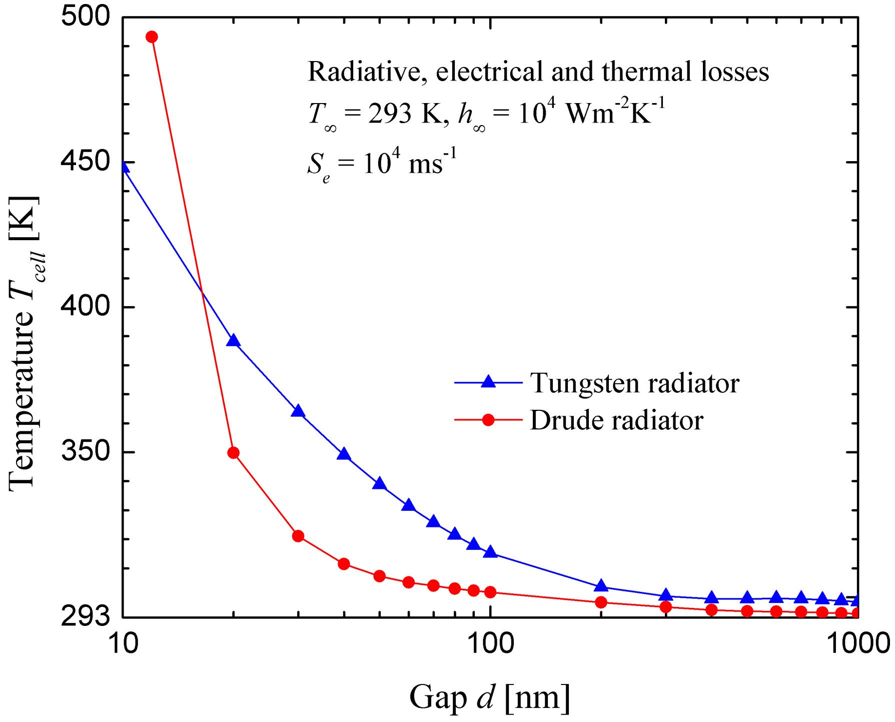

Figure S.2. Equilibrium cell temperature as a function of the vacuum gap thickness for tungsten and Drude radiators.

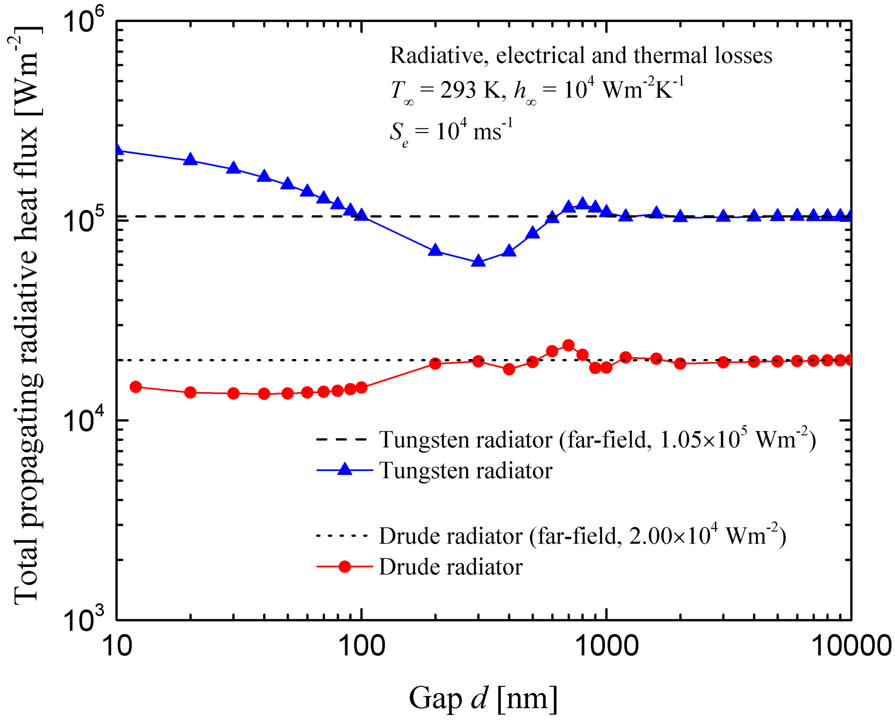

Figure S.3. Total propagating radiative heat flux absorbed by the cell as a function of the vacuum gap thickness for tungsten and Drude radiators.

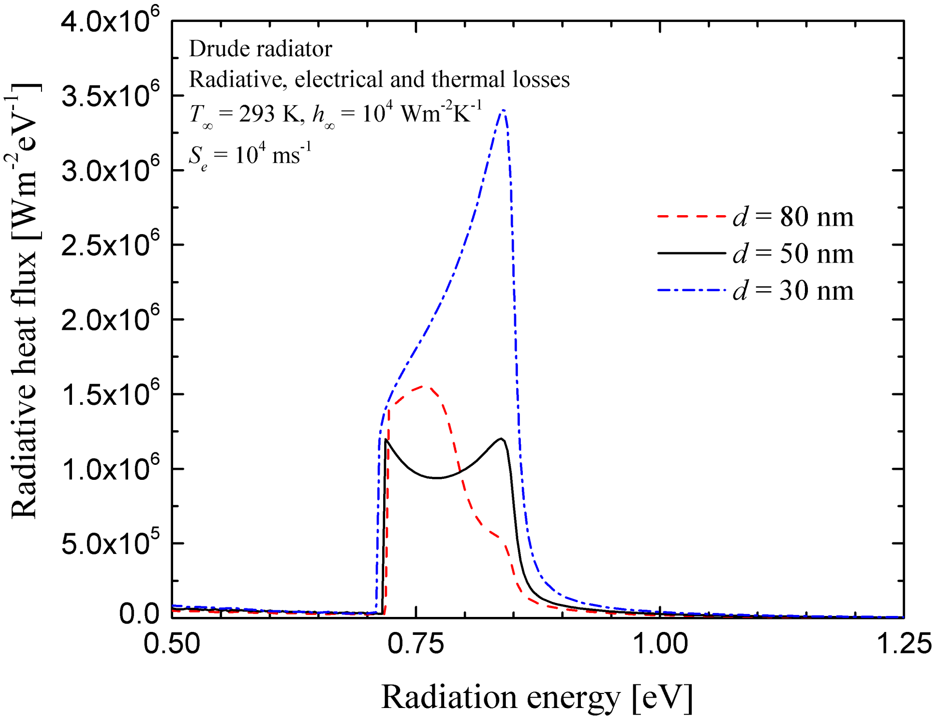

Figure S.4. Spectral distribution of radiative heat flux at the surface of the cell ($z = Z_2$) for vacuum gap thicknesses of 30, 50 and 80 nm (Drude radiator).

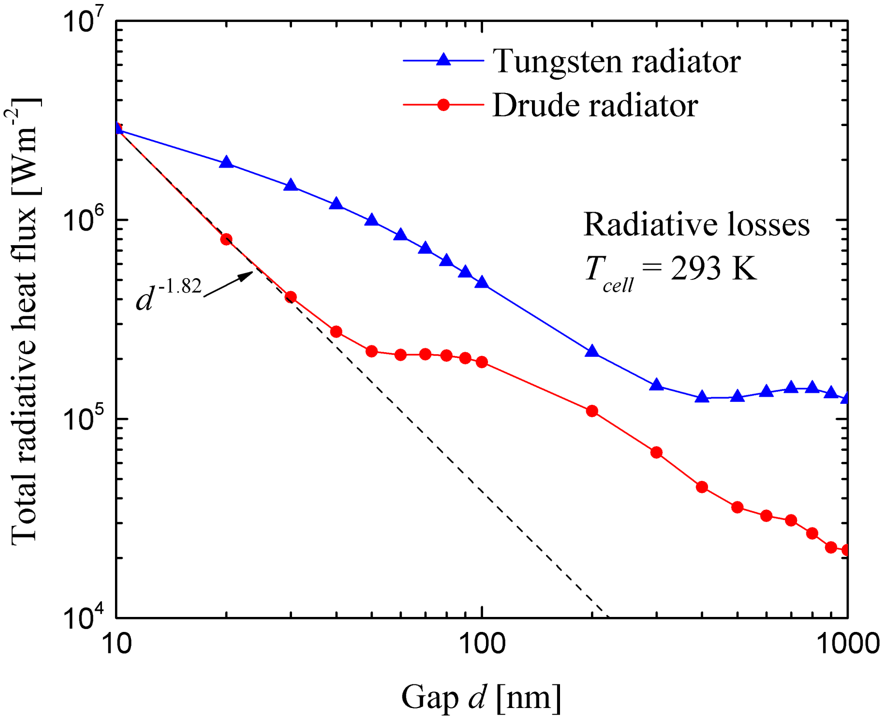

Figure S.5. Total radiative flux absorbed by the cell as a function of the vacuum gap thickness for tungsten and Drude radiators.

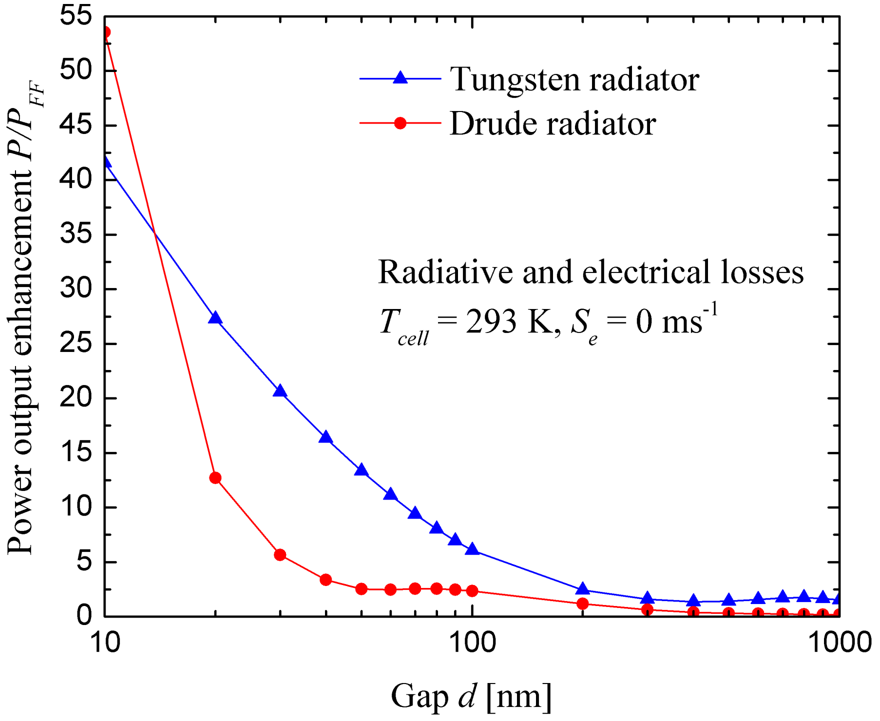

Figure S.6. Power output enhancement as a function of the vacuum gap thickness when neglecting surface recombination velocity and thermal losses for tungsten and Drude radiators.

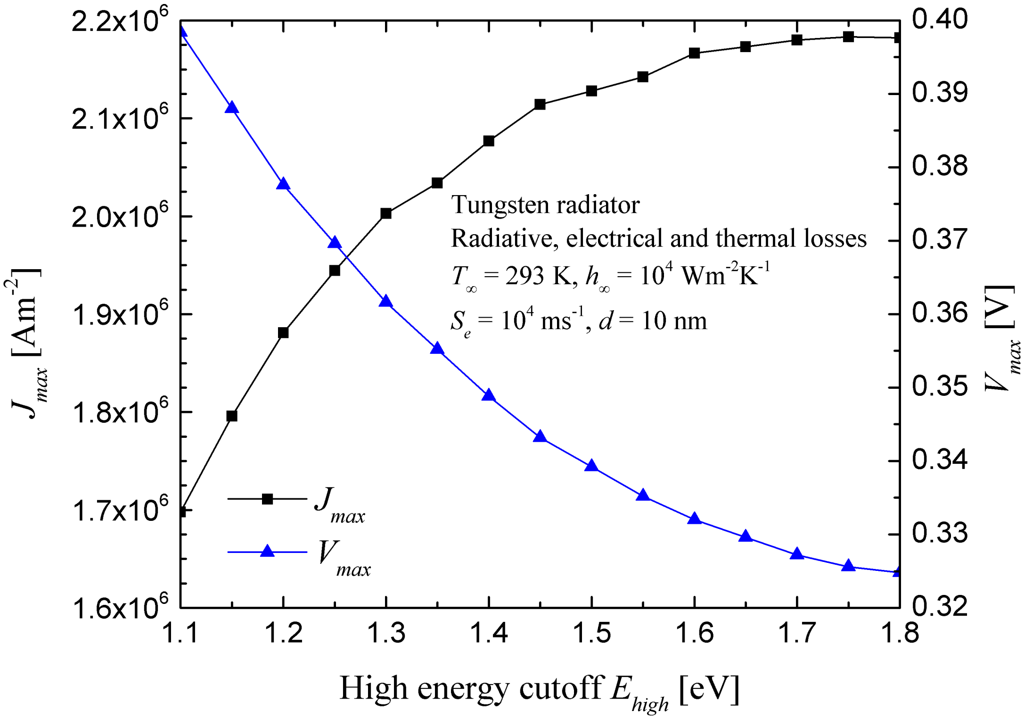

Figure S.7. Photocurrent J_{max} and potential V_{max} at maximum power output as a function of the high energy cutoff E_{high}.

Figure S.7 shows photocurrent J_{max} and voltage V_{max} at the maximum power output as a function of the high energy cutoff E_{high}. As expected, J_{max} increases as E_{high} increases due to a larger number of EHPs generated. Conversely, V_{max} decreases as E_{high} increases because of an increasing thermalization heat source and consequently a rise in temperature leading to a larger dark current.

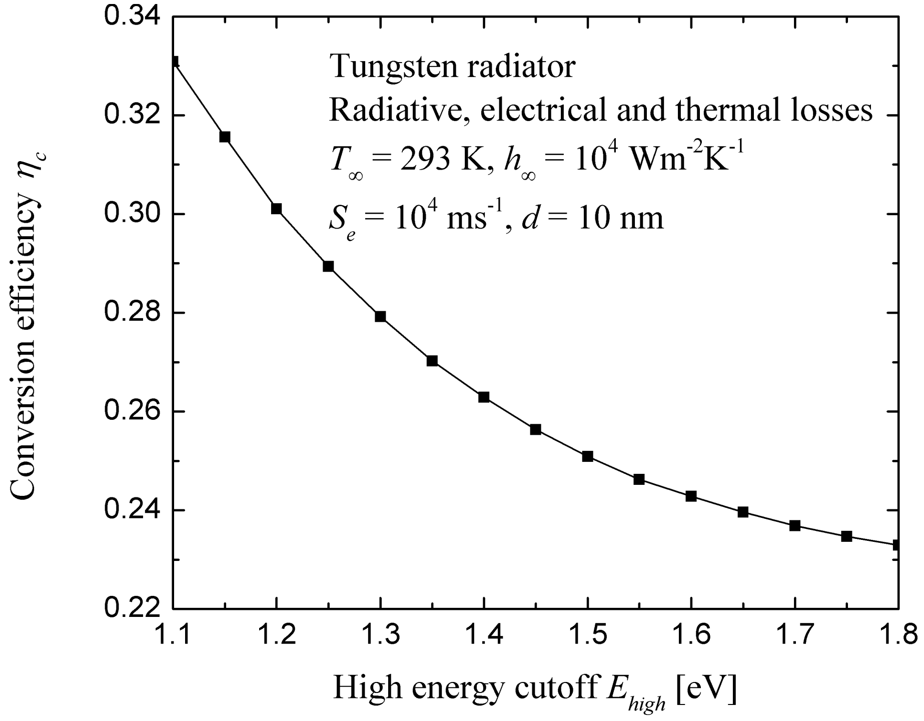

Figure S.8. Conversion efficiency as a function of the high energy cutoff E_{high} for a tungsten radiator.
